# Assessment of Feasibility of the M2 Macrophage-Based Adoptive Gene Transfer Strategy for Osteoarthritis with a Mouse Model

**DOI:** 10.3390/cells14141067

**Published:** 2025-07-11

**Authors:** Matilda H.-C. Sheng, David J. Baylink, Charles H. Rundle, Kin-Hing William Lau

**Affiliations:** 1 VA Loma Linda Healthcare System, Jerry L. Pettis Memorial VA Medical Center, Loma Linda, CA 92357, USA; charles.rundle@va.gov (C.H.R.); william.lau@va.gov (K.-H.W.L.); 2 Departments of Medicine and Biochemistry, Loma Linda University School of Medicine, Loma Linda, CA 92354, USA; dbaylink@llu.edu

**Keywords:** macrophages, inflammation, IL-1Ra, osteoarthritis, adoptive gene transfer, treatment

## Abstract

Current osteoarthritis (OA) therapies fail to yield long-term clinical benefits, due in part to the lack of a mechanism for the targeted and confined delivery of therapeutics to OA joints. This study evaluates if M2 macrophages are effective cell vehicles for the targeted and confined delivery of therapeutic genes to OA joints. CT bioluminescence in vivo cell tracing and fluorescent microscopy reveal that intraarticularly injected M2 macrophages were recruited to and retained at inflamed synovia. The feasibility of an M2 macrophage-based adoptive gene transfer strategy for OA was assessed using IL-1Ra as the therapeutic gene in a mouse tibial plateau injury model. Mouse M2 macrophages were transduced with lentiviral vectors expressing IL-1Ra or GFP. The transduced macrophages were intraarticularly injected into injured joints at 7 days post-injury and OA progression was monitored with plasma COMP and histology at 4 weeks. The IL-1Ra-expressing M2 macrophage treatment reduced plasma COMP, increased the area and width of the articular cartilage layer, decreased synovium thickness, and reduced the OARSI OA score without affecting the osteophyte maturity and meniscus scores when compared to the GFP-expressing M2 macrophage-treated or PBS-treated controls. When the treatment was given at 5 weeks post-injury, at which time OA should have developed, the IL-1Ra-M2 macrophage treatment also reduced plasma COMP, had a greater articular cartilage area and width, decreased synovial thickness, and reduced the OARSI OA score without an effect on the meniscus and osteophyte maturity scores at 8 weeks post-injury. In conclusion, the IL-1Ra-M2 macrophage treatment, given before or after OA was developed, delayed OA progression, indicating that the M2 macrophage-based adoptive gene transfer strategy for OA is tenable.

## 1. Introduction

Osteoarthritis (OA) is a painful, debilitating joint disease characterized by acute and chronic intraarticular inflammation, articular cartilage deterioration, and degenerative changes to periarticular and subchondral bone structures [1]. About 12% of OA is caused by acute trauma to the joint and is referred to as posttraumatic OA or PTOA [2]. The prevalence and progression of OA/PTOA are influenced by a combination of biomechanical, metabolic, and demographic factors, including occupational workload, high-impact sports participation, a history of joint injuries, obesity, and sex-related differences in joint anatomy and hormonal regulation [3,4]. OA/PTOA is the most common degenerative joint disease, afflicting >7% of people globally (~528 million people) with an economic impact (including direct and indirect costs) ranging from 1 to 2.5% gross national product in countries with established market economies [5].

Early-stage OA/PTOA is typically associated with an acute synovial inflammation (synovitis) followed by chronic low-grade intraarticular inflammation, including synovitis, throughout the disease progression. Synovitis activates synovial macrophages (MΦs) to secrete pro-inflammatory cytokines, which rapidly enhance chondrocyte apoptosis and the release of matrix proteinases that leads to the degradation of articular cartilage and subchondral bone matrices [6]. Synovitis contributes to effusion, pain, chondrocyte apoptosis, and persistent breakdown of articular cartilage [7], and promotes the growth and maturation of osteophytes [8] that irritate surrounding soft tissues, interfere with joint movement, put pressure on nearby nerves, and cause pain and discomfort. Accordingly, synovitis correlates positively with the severity of OA symptoms and the extent of articular cartilage destruction [9], and is thereby a major etiology/pathology of OA/PTOA.

Damaged articular cartilage does not heal by itself, as articular cartilage is poorly vascularized and its lesions often do not penetrate into subchondral bone for the access to bone marrow stem/progenitor cells needed for the repair [10]. Patients with mild OA are treated with symptom-modifying therapies primarily for pain management and inflammation reduction [7], but most patients found these therapies unsatisfactory [11,12]. Patients with the severe disease may require surgical interventions, and the results of which are often disappointing, as they are unable to restore the normal cartilaginous surface as well as suffering from poor integration into the existing articular cartilage [13]. Non-surgical therapies under development are generally categorized as anti-catabolic or anabolic. Most anti-catabolic therapies aim to suppress the production or activity of pro-inflammatory cytokines [7,14,15,16,17,18] or to reduce inflammation and cartilage degradation with caspase inhibitors, MMP inhibitors, or anti-inflammatory cytokines [7,19,20,21]. Anabolic therapies often utilized mesenchymal stem cells (MSCs), chondrocytes, protein/scaffold, or gene transfer strategies to locally deliver a chondrogenic growth or transcription factor or gene to promote cartilage regeneration [22,23]. However, these strategies fail to repair the damaged articular cartilage and yield only marginal clinical benefits [24]. One of the potential principal reasons might be the lack of an effective targeting mechanism that could provide confined and sustained delivery of the therapeutic or gene to the OA/PTOA joint.

Synovitis does not produce overt systemic inflammatory symptoms [25], but it is still capable of recruiting, activating, and promoting polarization of MΦs [26,27,28]. Traditionally, activated MΦs are classified into two major phenotypes (M1 and M2), based initially on their actions on inflammation [29,30], in that M1 MΦs are pro-inflammatory; whereas M2 MΦs are anti-inflammatory [31]. However, MΦs are highly plastic cells and can switch phenotype in response to environmental cues [30]. They also exhibit a high degree of heterogeneity. For example, M2 MΦs exist in at least three major subtypes with different sources, functions, surface receptors, and signaling pathways [32]. The functional roles of each phenotype are also context-dependent and vary with diseases [30]. Thus, the M1/M2 paradigm of MΦ classification is probably overgeneralized, but this simple M1/M2 paradigm of MΦ classification does provide a useful framework, particularly for selected immune responses. Accordingly, we opted to use this simple classification paradigm in this study to facilitate interpretation of the results.

In the context of OA, M1 MΦs play key roles in articular cartilage destruction, osteophyte formation, and anti-chondrogenic process [33,34]. The number of M1 MΦs in the synovium correlated positively with synovial angiogenesis [26] and OA progression [35]. Conversely, M2 MΦs are involved in tissue repair [36]. In the healthy joint, synovial MΦs predominantly show the M2 phenotype. During the acute inflammation phase, polarization of synovial MΦs moves towards the M1 phenotype, but it shifts back to the M2 phenotype at the latter chronic inflammation phases [37,38]. The ratio of M2 to M1 MΦs is inversely proportional to OA severity, indicating a strong correlative relationship between MΦ polarization and OA progression [37]. Accordingly, we speculate that M2 MΦs could provide a unique cell vehicle for the confined and targeted delivery of a therapeutic gene to inflamed OA joints at where an anti-inflammatory therapy is most needed.

This study represents an initial assessment of the feasibility of an M2 MΦ-based adoptive gene transfer strategy for OA/PTOA, using interleukin 1 receptor agonist (*IL-1Ra*) as the therapeutic gene in a validated mouse closed intraarticular tibial plateau compression loading-induced injury model of OA/PTOA [39]. We chose this mouse model because it is noninvasive, does not involve surgically opening the joint, preserves the integrity of the synovial environment, avoids manipulation of the articular tissues, and eliminates any issues concerning potential infection due to the surgical procedure. This approach causes substantial injuries to the synovium, meniscus, and articular cartilage surface of the tibial plateau but without fractures. The tibial plateau was chosen as the injury site, because it has a thicker articular cartilage layer than do other joints [40] and it is one of the most common sites of traumatic insults at lower extremities in PTOA patients [41]. We purposely employed a compression loading that would not cause tibial plateau fractures to avoid any confounding issues caused by the fracture-healing process during the interpretation of the results. The severity of OA/PTOA induced by this model was usually less severe than that induced by other more invasive OA/PTOA animal models. However, we believe that this model should be useful for the testing of the feasibility of the M2 MΦ-based platform for an adoptive gene transfer therapeutic strategy for OA/PTOA, which is our primary objective. In this study, we demonstrate for the first time that a single intraarticular injection of M2 MΦs expressing *IL-1Ra* reduced many symptoms of OA, and thereby supports the feasibility of M2 MΦ-based adoptive gene transfer strategies for OA/PTOA.

## 2. Materials and Methods

### 2.1. Animals

Male C57BL/6J mice were purchased from the Jackson Laboratory (Bar harbor, ME, USA). Animals were housed in groups of 4 per cage at 20 °C under a 12 h light/dark cycle and provided with water and regular rodent chow ad libitum. Male mice at 10 weeks of age were used in this study to demonstrate the feasibility of the M2 MΦ-based strategy for OA. Once accomplished, results will be confirmed in female mice in the future. Male global GFP transgenic (UBC-GFP) C57BL/6J (also from the Jackson Laboratory) at 10 weeks of age were used to histologically confirm the location of implanted cells in the knee joint with fluorescence-based histology. All animal protocols were approved by the Institutional Animal Care and Use Committee (IACUC) of the VA Loma Linda Healthcare System, the IACUC of the Loma Linda University, and the Animal Care and Use Review Office (ACURO) of the US Army Medical Research and Materiel Command of the Department of Defense. In conducting research using animals, the investigators adhered to the Animal Welfare Act Regulations and other Federal statutes relating to animals and experiments involving animals.

### 2.2. Polarization and Expansion of Marrow-Derived M2 MΦs

Bone marrow cells, isolated from both limbs of C57BL/6J mice, were cultured on untreated culture dishes in GIBCO high-glucose DMEM (ThermoFisher Scientific, Riverside, CA, USA) supplemented with 10% fetal bovine serum, in the presence of 25 ng/mL of mCSF (Peprotech, Cranbury, NJ, USA) for 6 days. One half of the culture medium was replaced with fresh medium every 2 to 3 days. The cultures were rinsed once with fresh medium after the removal of unattached marrow cells, and were then treated with 25 ng/mL mCSF and 20 ng/mL IL-4 (Peprotech, Cranbury, NJ, USA) until confluence to promote M2 MΦ polarization and expansion [42]. The expanded M2 MΦs were immediately used or kept frozen in liquid nitrogen.

### 2.3. Lentiviral Vector Preparations

Primary MΦs are notoriously difficult to transfect even with viral vectors [43]. However, we previously reported that Gr1^+^ M2 MΦs can be effectively transduced with lentiviral-based vectors to express 25-hydroxyvitamin D_3_ 1α-hydroxylase [44]. To generate the lentiviral vector expressing *IL-1Ra*, the mouse *IL-1Ra* gene was molecularly cloned by PCR with DNA of the *IL-1Ra* cDNA clone (Origene, Rockville, MD, USA) as the template and a PCR primer set consisting of the forward primer (5′-AAA AGC GAT CGC TGT TTA GC-3′) and the reverse primer (5′-AAA ACA AAT TTG AGG CCT CG-3′). The resulting PCR product contained the coding sequence of *IL-1Ra* with a poly-A tail, the added AsisI and PMEI restriction sites (needed for subsequent subcloning), and a Kozak sequence. The resulting *IL-1Ra* cDNA product was then subcloned into the pRRLsin-cPPT-SFFV-X-hPGK-*GFP*wpre lentiviral expression vector (kindly provided by Dr. X.B. Zhang of the Loma Linda University) to produce the bicistronic pSFFV-*IL-1Ra*-hPGK-*GFP*wpre (pSFFV-*IL-1Ra-GFP* or simply pSFFV-*IL-1Ra*) lentiviral expression vector. The parental pRRLsin-cPPT-SFFV-GFP-hPGK-Xwpre (pSFFV-*GFP*) lentiviral expression vector was used as controls for comparison. DNA sequence of the 527-bp *IL-1Ra* insert of the constructed pSFFV-*IL1Ra*-*GFP* plasmid was confirmed by DNA sequencing (McLab, South San Francisco, CA, USA).

To generate lentiviral vectors, HEK293T cells were plated onto a 10 cm dish at a density of 7–8 × 10^6^ cells per dish overnight prior to the viral transfection by the calcium phosphate co-precipitation method. Briefly, HEK293T cells were incubated with the pSFFV-*IL-1Ra-GFP* or the pSFFV-*GFP* viral plasmid along with the VSV-G and Pax2 capsid plasmids in the presence of calcium phosphate. The virus medium of transfected cells, which contained lentiviral particles, was collected after 48 h. The viral particles were collected by centrifugation and were used immediately or stored frozen at −80 °C until use.

### 2.4. Lentiviral Vector Transduction of Primary Murine M2 MΦs

The attached M2 MΦs were plated into 6-well plates with a density of 1 to 2 × 10^6^ per well in the presence of mCSF (25 ng/mL) and IL-4 (20 ng/mL) for 1–2 days before transduction in cultures. The transduction was performed with simple incubation without low-speed centrifugation (or “spinfection”). Transduction efficiency was determined by the expression levels of GFP in the transduced cells. The viral particles were then added into the cultures in the presence of protamine sulfate and mCSF/IL-4 for 16 h. The cell medium was replaced with freshly prepared medium containing mCSF/IL-4 for an additional 3 to 4 days for expansion before use.

### 2.5. Bioluminescence-Based In Vivo Tracking of the Fate of Intraarticularly Injected M2 MΦs in Injured Knee Joints

We used the IVIS Spectrum CT system (Perkin-Elmer, Waltham, MA, USA) to monitor the fate and duration-of-stay of the injected M2 MΦs in vivo, because this technology provides high-sensitivity in vivo imaging of fluorescence or bioluminescence in small animals. We chose to use the bioluminescence approach (i.e., luciferase-expressing M2 MΦs) to monitor the location and distribution of the injected M2 MΦs, because the IVIS Spectrum CT system shows much higher sensitivity for bioluminescence than fluorescence. Briefly, luciferase-expressing M2 MΦs were generated by transducing M2 MΦs with the pLenti-CMV-Puro-*LUC* (Addgene, Watertown, MA, USA, cat. # 17477), a commercial luciferase-expressing lentiviral vector, as described above. A total of 0.5 million of *Luc*-expressing M2 MΦs were intraarticularly injected into each injured joint of a group of five male 10-week-old C57BL/6J mice at 7 days after the tibial plateau injury. At 2, 7, and 14 days after the cell injection, each mouse received an i.p. injection of 150 µg/g body weight of D-luciferin (GoldBio, St Louis, MO, USA). The location and relative intensity of the bioluminescence were monitored with the Perkin-Elmer IVIS Spectrum CT imaging system.

### 2.6. Flow Cytometry

Flow cytometric analysis was performed in an FACS Aria II (BD Biosciences, San Jose, CA, USA) with a 488 nm laser. For identification of M2 MΦs, marrow-derived MΦs, after 3 days of mCSF alone or mCSF and LPS, or mCSF and IL-4 co-treatments, were stained for the M2 MΦs cell surface marker CD206 by incubating with CD206-PE monoclonal antibody (ThermoFisher Scientific, Riverside, CA, USA) for 30 min at room temperature prior to flow cytometry analysis. Thirty thousand events were collected for each analysis.

### 2.7. A Mouse Tibial Plateau Injury Model

A validated closed intraarticular tibial plateau compression loading-induced injury model of OA/PTOA [39] was used in this study. The detailed procedure has been described previously [39,45]. Briefly, the injury was produced on the intraarticular tibial plateau of the right knee joints of male 10-week-old C57BL/6J mice under isoflurane inhalation anesthesia. The right knee was positioned on a support attached to the load cell of an Instron servohydraulic mechanical tester. The excursion of a blunt indenter blade was set on the top of the tibial plateau, and an impact force was applied under a defined force of 55 N at a speed of 200 N/s. This impact force effectively caused injuries to the synovium, meniscus, and articular cartilage surface, but did not create fractures. These injuries consistently (seen in >99% of injured joints) led to progressive OA/PTOA development with clear histological evidence for OA development at 5 weeks post-injury [39,45,46].

### 2.8. The IL-1Ra-Expressing M2 MΦ-Based Treatment Strategy for OA/PTOA

Briefly, the intraarticular tibial plateau injury was created on the right knee joint of each mouse. At 7 days post-injury, 0.5 to 1.0 million of *IL-1Ra* (or *GFP*-)-expressing M2 MΦs were injected into the intraarticular space of the injured knee joints. We chose 7 days post-injury as the administration time, because MΦ-based therapy initiated at 8 or 9 days post-injury recovered better in a rat spinal cord injury model [47], and the 7-day time point represents the transition point from the early acute inflammation phase to the later tissue-repairing phase, at which time the MΦ polarization begins to shift from the M1 to M2 phenotype. We reason that this would be beneficial for the recruitment and retention of M2 MΦs at the inflamed joint. At each indicated time point, the animals were sacrificed. The progression and severity of OA in each injured joint were monitored by histology as described below.

### 2.9. Plasma Cartilage Oligomeric Matrix Protein (COMP)

Blood samples were collected from each mouse at sacrifice, and plasma samples were prepared. The plasma level of COMP was measured with a commercial ELISA kit for COMP (Aviva Systems Biology, San Diego, CA, USA).

### 2.10. Histology

For frozen sections, the knee joint along with the tibia and femur, after fixation with Zamboni’s fixative for 12 h at 4 °C, was embedded in an embedding medium (SECTION-LAB, Co., Ltd., Hiroshima, Japan). Frozen thin sections (3 µm thick) were prepared with a Leica cryostat and mounted onto a plastic film (Cryofilm type IIC, SECTION-LAB, Hiroshima, Japan). The frozen sections were evaluated for GFP signal or stored in 100% ethanol at −20 °C until histological evaluation. For paraffin sections, the injured right joints (including distal femoral and proximal tibial ends) were fixed with 10% cold neutral buffered formalin for 3 to 4 days, rinsed with PBS, and stored in PBS. The fixed joints were decalcified, dehydrated, and embedded in paraffin by standard procedures. Serial 5 µm sagittal thin sections corresponding to the injured tibial plateau region were cut from the medial side, deparaffinized, followed by rehydration with graded ethanol, and stained with 0.4% toluidine blue in acetate buffer (pH 4). The average width and area of the articular cartilage layer around the impacted tibial plateau on serial sagittal thin sections of each joint were measured with the OsteoMeasure™ system (SciMeasure Analytical Systems, Decatur, GA, USA, latest v. 4.1.0.2) using the software originally designed for measurements of the average distance between the double tetracycline-labeled surfaces and the osteoid area, respectively. [These are two dynamic bone formation histomorphometry parameters.] The average of the values on three serial sagittal sections of each joint was reported.

### 2.11. Histology-Based OA Severity Scoring Systems

The relative severity of OA was determined in a blinded fashion according to the OARSI OA Articular Cartilage Histopathology Assessment method [48], which was an articular cartilage histology-based scoring system assessing the relative severity of articular cartilage deterioration using a scale from 0 to 6, with 6 being the most severe and 0 being having no OA. The relative severity in meniscal pathology was evaluated in a blinded fashion with a histology-based meniscus scoring system [49], according to three criteria: (1) surface integrity; (2) cellularity (outer, inner, and superficial zone); and (3) toluidine blue staining distribution and intensity of the menisci in the injured joints. Total scores from both anterior and posterior menisci were added together. Grade 0 = 0–4; Grade 1 = 5–9; Grade 2 = 10–14; Grade 3 = 15–19; Grade 4 = 20–24; Grade 5 = 25–29; Grade 6 = 30–35. The extent of osteophyte formation was assessed using an osteophyte maturity scoring system developed by Jono et al. [50]: a score of 0 = no osteophytes; 1 = periosteal synovial hypertrophy; 2 = mixed periosteal synovial hypertrophy and cartilage extending proximally from the growth plate; 3 = predominantly cartilage; 4 = mixed cartilage and bone with active vascular invasion and endochondral ossification; and 5 = predominantly bone. We chose the osteophyte maturity scoring system to assess osteophyte development, because this scoring system evaluates osteophyte formation by comparing the tissue nature within the osteophyte structure [51]. Osteophyte is formed first as cartilaginous tissues that are progressively matured to bony tissues through endochondral ossification. We believe that comparison between cartilaginous vs. bony tissues within the osteophyte structure would be a more accurate index of osteophyte formation than osteophyte size.

### 2.12. Statistical Analyses

Statistical analysis was performed with Systat11 software (Systat Inc., Richmond, CA, USA) or with GraphPad Prism version 7.03 (GraphPad Software, San Diego, CA, USA). Normal distribution and variance homogeneity of the data were first confirmed with the Shapiro–Wilk test and the Levene’s test, respectively. Statistical significance of the differences was analyzed with one-way analysis of variance (ANOVA) followed by Tukey’s post hoc test (for multiple groups) or with two-tailed Student’s *t*-test (for two groups). Results are shown as mean ± SEM. It was considered significant when *p* < 0.05.

## 3. Results

### 3.1. Lentiviral-Transduced M2 MΦs Secreted Large Amounts of IL-1Ra Protein

**Figure 1:**
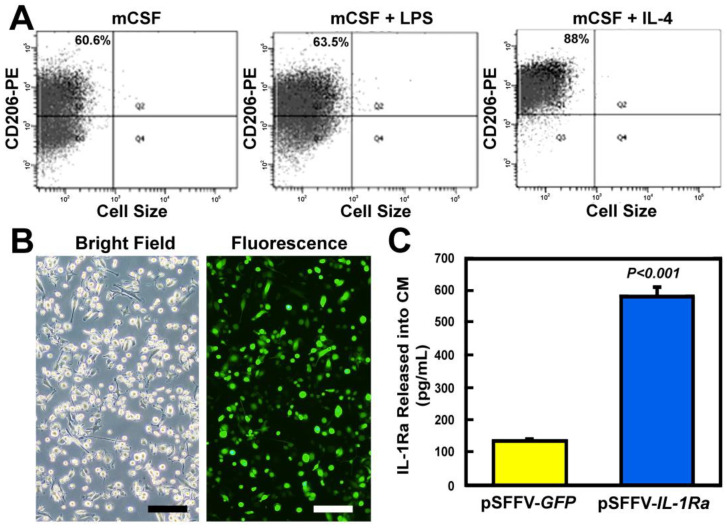
Levels of IL-1Ra protein secreted into CM by mCSF/IL-4-expanded pSFFV-*IL-1Ra*-transduced M2 MΦs in cultures. (**A**) Florescence-assisted cell sorting (FACS) analysis of murine M2 MΦs for CD206, a biomarker specific for M2 MΦs, after expansion with mCSF alone (**left**), expansion and M1 polarization with the mCSF and LPS co-treatment (**middle**), and expansion and M2 polarization with the m-CSF and IL-14 co-treatment (**right**). [Each expanded and polarized cell population was also sorted for CD11b and F4/80, and each was >97 and >98% positive for CD11b and F4/80, respectively, indicating that there were indeed MΦs.] (**B**) A representative bright-field (**left**) and fluorescent (**right**) photomicrograph of GFP expression in a representative culture of IL-1Ra/GFP-expressing murine M2 MΦs. The transduction efficiency in this experiment, judged by GFP expression, was estimated to be ~70%. Scale bars = 20 µm. (**C**) The CM of the transduced cells after 72 h in culture was collected and the relative IL-1Ra protein level in CM was measured by ELISA. Results are shown as mean ± SEM with *n* = 4 per group. Statistical significance of the difference between the two groups was determined with two-tailed Student’s *t*-test.

To expand the M2 MΦ population, marrow-derived M2 MΦs were co-treated with mCSF and IL-4 for 3 days. Quantitative flow cytometry against CD206, an M2 MΦs cell surface marker [52], indicates that the purity of the expanded M2 MΦs was ~90% (Figure 1A, right panel). For comparison, for the expanded MΦs treated with mCSF alone without polarization cytokines, the basal percentage of CD206 positive cells was ~61% (left panel), and the percentage after incubation under condition for M1 polarization (i.e., with the mCSF and LPS co-treatment) was not much different from the basal (i.e., ~64% vs. ~61%). The M2 MΦs were transduced (using an MOI of 2–10) with pSFFV-based bicistronic lentiviral vectors expressing both *IL-1Ra* and *GFP* (as a marker gene). The transduction efficiency, judged by GFP expression, was between 70 and 90% (Figure 1B). The amount of IL-1Ra protein released into conditioned media (CM) by transduced M2 MΦs was ~4-fold greater than that by the pSFFV-*GFP*-transduced control M2 MΦs (Figure 1C), confirming that the genetically modified M2 MΦs produced and released into CM substantial amounts of IL-1Ra protein.

### 3.2. Retention of Intraarticularly Injected M2 MΦs in the Injured Joint

**Figure 2:**
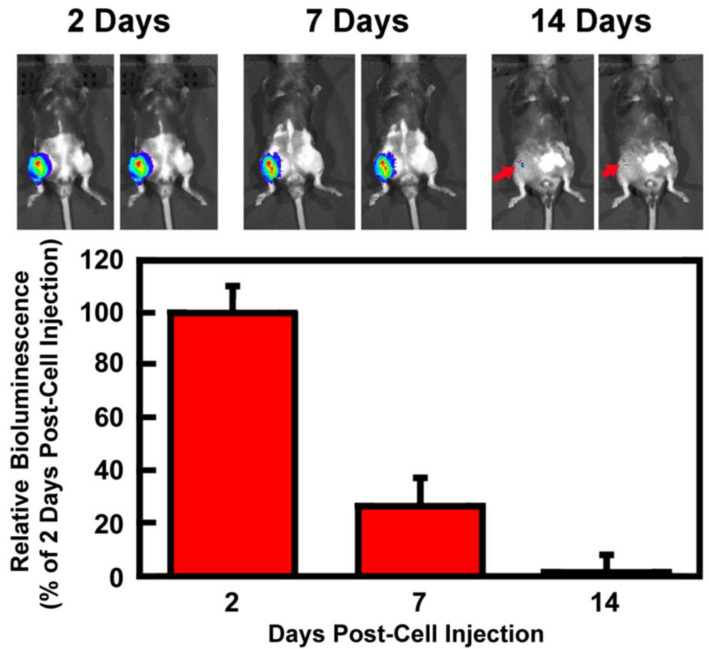
Temporal and spatial tracing of the luciferase-expressing M2 MΦs in the injured knee joint in vivo after intraarticular injection. Intraarticular tibial plateau injury was created on the right knee joint of 10-week-old C57BL/6J mice. At 7 days post-injury, *Luc*-expressing M2 MΦs were injected intraarticularly. At 2, 7, and 14 days after the cell injection, D-luciferin was injected i.p. into each mouse. The tracking of the injected *Luc*-expressing M2 MΦs was performed with Perkin-Elmer IVIS CT bioluminescence in vivo imaging system. Top is the CT bioluminescence image of two representative mice at each time point. Bottom shows the relative levels of bioluminescence emitted, as a percentage of bioluminescence emitted on day 2. Results are shown as mean ± SEM (*n* = 5).

We applied an in vivo bioluminescence imaging tracing approach (using the Perkin-Elmer IVIS CT imaging system) to assess the temporal and spatial distribution profiles of the injected M2 MΦs in the injured knee joint after a single intraarticular injection of ~0.5 million luciferase-expressing M2 MΦs, which were produced by transducing M2 MΦs with a commercial pLenti-CMV-Puro-*LUC* lentiviral vector, at 7 days post-injury (i.e., after the peak of the acute synovial inflammation phase). At 2, 7, and 14 days after the cell injection, each animal received an i.p. injection of D-luciferin for the detection of luciferase activity. The temporal and spatial distribution profiles of bioluminescence emitted by luciferase-expressing cells in vivo were monitored. Figure 2 shows that at 2 days after cell injection, the injected luciferase-expressing cells were found exclusively in the injected right knee joint with no detectable luciferase activity elsewhere. At 7 days post-injury, ~26% of luciferase activity remained exclusively at the injured joint. At 14 days post-injection, only~1% of the bioluminescence (red arrows) remained, indicating that a low but still substantial number of *Luc*-expressing M2 MΦs were retained exclusively at the injured joint even after 2 weeks. Thus, this study confirms that the injected M2 MΦs were recruited to the inflamed joint with a duration-of-stay period of at least 14 days. It also suggests that a single injection might be sufficient to elicit desirable clinically relevant outcomes.

**Figure 3:**
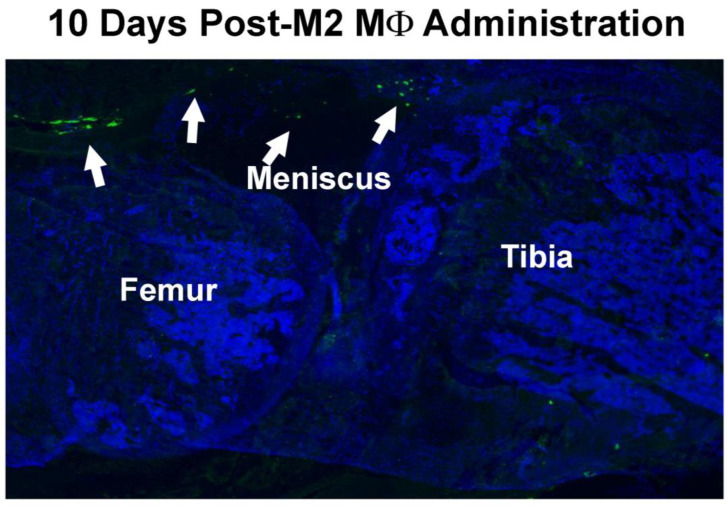
Recruitment and retention of intraarticularly injected M2 MΦs in the inflamed synovial membrane. A representative fluorescent photomicrograph of the location of *GFP*-expressing M2 MΦs in the injected joint. *GFP*-expressing M2 MΦs (isolated from *GFP* transgenic mice) were injected intraarticularly into the injured joint on day 4 post-injury and sagittal sections of the injured knee joint were examined by fluorescent microscopy 10 days later (i.e., 17 days post-injury).

To confirm the intracellular location of *GFP*-expressing M2 MΦs in the injured joint, *GFP*-expressing M2 MΦs, isolated from global *GFP* transgenic mice, were intraarticularly injected into the injured knee joint at 4 days post-injury and *GFP*-expressing M2 MΦs were identified on thin sagittal sections by fluorescence histomorphometry at 10 days after the cell injection (i.e., 17 days post-fracture). The location of *GFP*-expressing cells was found to be restricted to the synovium (Figure 3), confirming that locally injected M2 MΦs were recruited to and retained at the inflamed synovium.

### 3.3. M2 MΦs-Based Adoptive IL-1Ra Gene Transfer Strategy Was Effective in Reducing Plasma Levels of COMP

**Figure 4:**
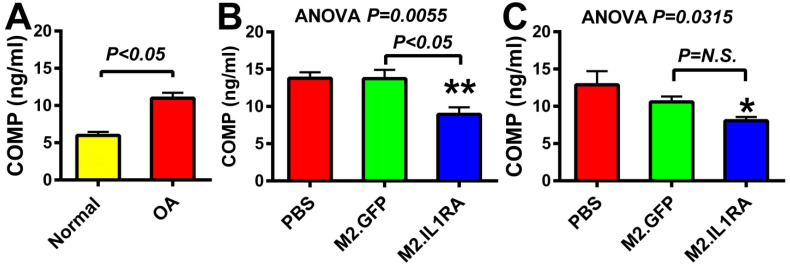
Plasma levels of COMP in mice treated with M2 MΦs expressing *IL1Ra* at 1 or 5 weeks post-tibial plateau injury. (**A**) Plasma COMP levels of normal healthy mice (left) or untreated OA mice at two weeks post-tibial plateau injury. Statistical significance was determined by two-tailed Student’s *t*-test. (**B**) Plasma COMP levels at 4 weeks post-injury in mice receiving a single injection of *IL-1Ra*- or *GFP*-expressing M2 MΦs at 1 week post-injury. (**C**) Plasma COMP levels at 8 weeks post-injury in mice receiving a single injection of *IL-1Ra*- or *GFP*-expressing M2 MΦs at 5 weeks post-injury. N = 6–7 mice per test group. Statistical significance in (**B**,**C**) was determined by one-way ANOVA followed by Tukey’s post hoc test. * *p* <0.05 and ** *p* < 0.01 compared to the PBS control group. *P* = N.S. (not significant), when *p* > 0.05.

As an initial test of the feasibility of the M2 MΦ-based adoptive gene transfer strategy for OA, a single dose of 0.5 to 1.0 million of *IL-1Ra*-expressing or *GFP*-expressing M2 MΦs were intraarticularly injected into the injured joint on either 7 days or 5 weeks after the tibial plateau injury, and the plasma level of COMP was measured at 4 and 8 weeks post-injury, respectively. COMP is an extracellular matrix (ECM) glycoprotein critical for collagen assembly and ECM stability. In chondrocytes and synovial cells, COMP expression is activated by pro-inflammatory cytokines [53], and mechanistically, it is released into the joint fluid following injury and during the early stages of OA [54]. It is a well-established circulating biomarker of cartilage turnover that is associated with the destruction of cartilage, including articular cartilage and fibrocartilage (meniscus), and thereby is considered a useful circulating biomarker for the diagnosis and prediction of OA progression [55]. Accordingly, Figure 4A shows that the plasma level of COMP of a group of untreated 10-week-old male OA mice after two weeks of the intraarticular tibial plateau injury was elevated about two-fold compared to that of a group of age- and sex-matched normal healthy C57BL/6J mice, confirming that the injury to the tibial plateau increased articular cartilage catabolism. Figure 4B shows that when the M2 MΦs were administered at 1 week post-injury, the *IL-1Ra*-M2 MΦ group treatment significantly reduced the circulating level of COMP by >30% compared to the *GFP*-M2 MΦs group or to the untreated (PBS-treated) group. When the cells were administered at 5 weeks post-injury, when OA should have been developed [39,45,46], the *IL-1Ra*-M2 MΦs treatment also significantly reduced plasma COMP levels by >25% compared to M2 MΦs alone and the untreated groups (Figure 4C). These findings are consistent with the premise that the *IL-1Ra* M2 MΦ2 therapy is effective in the suppression of OA-induced articular cartilage degradation, regardless of when it was initiated before or after OA is developed. However, the plasma COMP level in the M2 MΦ alone (i.e., *GFP*-M2 MΦs) group at either time point was not significantly different from the untreated (PBS) group. We interpret that although M2 MΦs have intrinsic anti-inflammatory actions [31], a single injection of M2 MΦs alone might be insufficient to suppress the OA-induced articular cartilage degradation.

### 3.4. M2 MΦs-Based Adoptive IL-1Ra Gene Transfer Strategy, When Administered at 1 Week Post-Injury, Was Effective in Reducing Severity of OA

**Figure 5:**
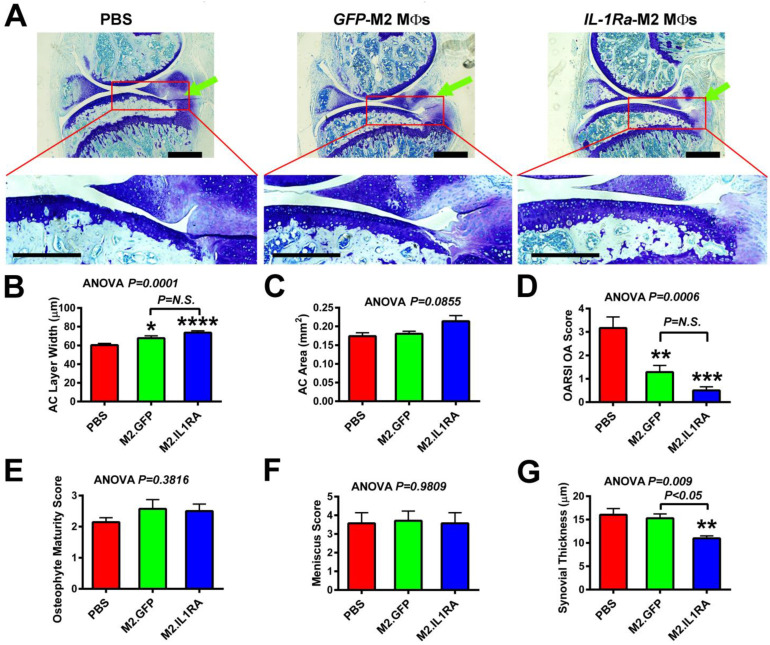
Effects of a single intraarticular injection of M2 MΦs expressing *IL1Ra* (M2.IL1RA) or *GFP* (M2.GFP), or PBS alone, at 1 week after the tibial plateau injury on OA progression. Tibial plateau injury was created on the right knee. The genetically modified M2 MΦs or PBS were injected intraarticularly at 1 week after injury. OA progression was monitored at 4 weeks post-injury by histology on sagittal thin sections (from the medial side). (**A**) Top, toluidine blue staining of cartilage of a representative injured joint of each treatment group. The green arrow indicates the site of compression loading on tibial plateau. Bottom, higher magnification of tibial plateau articular cartilage layer in each red box of the indicated knee joint. Scale bars = 250 µm. (**B**) Average articular cartilage (AC) layer width of tibial plateau; (**C**) average articular cartilage (AC) layer area; (**D**) OARSI OA score; (**E**) osteophyte maturity score; (**F**) meniscus score; and (**G**) average thickness of synovial layers. Results are shown as mean ± SEM, N = 6–7 per group. Statistical significance was determined by one-way ANOVA followed by Tukey’s post hoc test. * *p* < 0.05, ** *p* < 0.01, *** *p* < 0.001, and **** *p* < 0.0001, when compared with PBS (“no treatment”) group. *P* = N.S. (not significant), when *p* > 0.05.

We next analyzed the effect of the *IL-1Ra*-M2 MΦs therapy on the prevention of OA progression. Accordingly, a single dose of 0.5 to 1.0 million *IL-1Ra*-expressing M2 MΦs was injected intraarticularly into injured knee joints at 1 week post-injury. The progression of OA was analyzed 3 weeks later by histology. Figure 5A shows the toluidine blue staining of cartilage on thin sagittal sections of a representative PBS-treated, *GFP*-expressing M2 MΦ-, or *IL-1Ra*-expressing M2 MΦ-treated injured joint. The enlarged view (with a higher magnification) of the articular cartilage layer (identified by the red box) of the PBS-injected injured tibial joint exhibited evidence of abrasion of the usually smooth articular cartilage surface as well as focal discontinuity of the superficial zone, resulting in an irregular articular cartilage surface. It also exhibited a reduced cellularity of articular chondrocytes (i.e., fewer chondrocyte columns) presumably due to increased cell death and an increased clustering and disorientation of the chondrocyte columns. These well-known characteristics of OA/PTOA articular cartilage are also the chosen histological parameters used in the determination of the OARSI OA score [56]. The articular cartilage layer of the injured tibial plateau of *GFP*-expressing M2 MΦ-treated joints also showed similar degenerative changes as those seen in the PBS-treated injured tibial joint, albeit the degeneration changes were somewhat smaller. The smaller degenerative changes are not surprising and are also consistent with the premise that M2 MΦs per se have disease-modifying functions in OA/PTOA [38,57]. Conversely, there was no clear evidence of the surface erosion and/focal discontinuity of the superficial zone (i.e., with intact superficial and transitional zones) in the articular cartilage layer of the *IL-1Ra*-expressing M2 MΦ-treated tibial joints. The *IL-1Ra*-expressing M2 MΦ-treated tibial joints also had much greater articular chondrocyte cellularity without disorientation of the chondrocyte columns than the PBS-injected or *GFP*-expressing M2 MΦ-treated injured tibial joints. In addition, both the *GFP*-expressing and the *IL-1Ra*-expressing M2 MΦ treatments showed greater average articular cartilage width (Figure 5B), but the increase induced by the *IL-1Ra*-expressing M2 MΦs treatment was greater than that by the M2 MΦs alone control group. The increase in the average articular cartilage area did not reach the statistically significant level, perhaps due to the large variations in the measurement and/or the relatively small group size (Figure 5C). Nevertheless, these effects led to a significant reduction in the articular cartilage histology-based OARSI OA score in both the M2 MΦs alone and the *IL-1Ra*-M2 MΦ group compared to the PBS (untreated) group (Figure 5D). The effects in the *IL-1Ra*-M2 MΦs group appeared to be greater than those in the M2 MΦs alone group. That the M2 MΦs alone group showed an OA reduction effect is consistent with the previous observations that M2 MΦs have OA-modifying functions [38,57]. However, neither the *GFP*-M2 MΦ nor *IL-1Ra*- M2 MΦ treatment had significant effects on the osteophyte maturity score (Figure 5E) or meniscus score (Figure 5F), when compared to the untreated (i.e., PBS-treated) injured joints.

Synovial hyperplasia is a well-known hallmark for OA/PTOA, and IL-1Ra is a potent inhibitor of IL-1β-mediated synovitis [58]. Thus, we examined the effects of the therapy on synovial hypertrophy by measuring the average thickness of the synovial layers. As shown in Figure 5G, the average thickness of the synovium of untreated injured joints was increased from the usual 1 to 3 cell layers (e.g., ~2 to 6 µm in thickness) to multiple layers with an average thickness of >15 µm. The M2 MΦs treatment alone had no significant reduction effect on the average thickness of the synovium compared to the untreated joints, suggesting that despite the well-known anti-inflammatory actions of M2 MΦs, the M2 MΦs alone did not appear to be sufficient to decrease synovitis-induced swelling of the synovium (i.e., synovial hyperplasia) at the test dose. On the other hand, the *IL-1Ra*-M2 MΦs treatment significantly reduced the average thickness by >30% to ~11 µm, indicating that the *IL-1Ra*-M2 MΦ treatment is effective in the reduction in the OA-associated synovial inflammation. Based on the foregoing findings, we conclude that the *IL-1Ra*-M2 MΦ-based adoptive gene transfer strategy, when administered early, had OA protective effects and that the protective effect was greater than that of the M2 MΦs alone treatment.

### 3.5. M2 MΦs-Based Adoptive IL-1Ra Gene Transfer Strategy, When Administered After Development of OA, Was Effective in Halting or Delaying OA Progression

**Figure 6:**
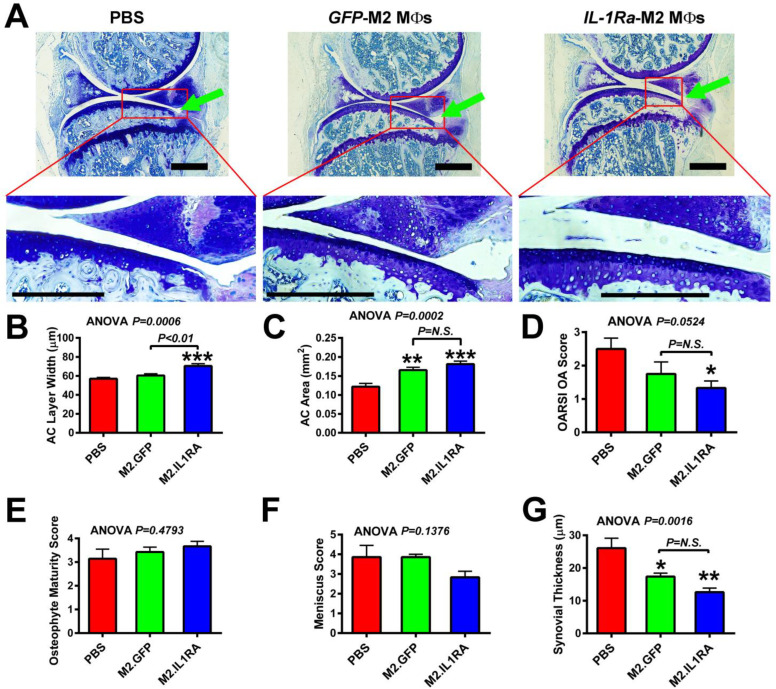
Effects of a single intraarticular injection of M2 MΦs expressing *IL1Ra* (M2.IL1RA) or *GFP* (M2.GFP), or PBS alone, at 5 weeks after the tibial plateau injury on OA progression. Tibial plateau injury was created on the right knee. Genetically modified M2 MΦs were injected at 5 weeks after injury, at which time OA should have been developed [39,45,46]. OA progression was determined 3 weeks later by histology on sagittal thin sections (from the medial side). (**A**) Top, toluidine blue staining of cartilage of a representative injured joint of each treatment group. Green arrow indicates the site of compression loading on the tibial plateau. Bottom, higher magnification of tibial plateau articular cartilage layer in each red box of the indicated knee joint. Scale bars = 250 µm. (**B**) Average articular cartilage (AC) layer width on the tibia of injured joints; (**C**) average articular cartilage (AC) layer area; (**D**) OARSI OA score; (**E**) osteophyte maturity score; (**F**) meniscus score; and (**G**) average thickness of the synovial layer. In each panel, results are shown as mean ± SEM, N = 6–7 per group. Statistical significance was determined by one-way ANOVA followed by Tukey’s post hoc test. * *p* < 0.05, ** *p* < 0.01, and *** *p* < 0.001, when compared with PBS (“no treatment”) group. *P* = N.S. (not significant), when *p* > 0.05.

We next determined whether this M2 MΦ-based adoptive gene transfer strategy is effective after OA has already been developed. Accordingly, M2 MΦs expressing *IL-1Ra/GFP* or *GFP* alone, or PBS, were administered into the injured joints at 5 weeks post-injury when OA should have already been developed [39,46,59], and the OA progression was monitored by histology three weeks later, i.e., 8 weeks post-injury (Figure 6A). The enlarged view of Figure 6A shows that when treatment was initiated at 5 weeks after injury and OA progression examined 3 weeks later, an erosion of the articular cartilage with discontinuity of the superficial zone was evident in PBS-injected joints but with a reduced relative severity compared to those seen in Figure 5A. Consistent with the presence of OA/PTOA-related degenerative changes in articular cartilage, there was also evidence for a reduced cellularity of articular chondrocytes as well as an increased clustering with the disorientation of chondrocyte columns in PBS-injected injured knee joints, indicating the presence of OA/PTOA-related degeneration of articular cartilage. Similarly, degenerative changes were detected in the *GFP*-expressing M2 MΦ-treated articular cartilage layer, but at a relatively less severity than the PBS-treated joints. However, the more salient observation is that treatment with *IL-1Ra*-expressing M2 MΦs yielded some protective actions on the degenerative changes in articular cartilage compared to the PBS- or *GFP*-expressing M2 MΦs treatment. The protective effects on articular cartilage seen in injured joints that received treatment at 5 weeks (Figure 6) appeared to be smaller than those on the articular cartilage of injured joints that were treated at 1 week post-injury (Figure 5A). The M2 MΦs treatment alone had no significant effects on the average articular cartilage width (Figure 6B) but significantly increased (by ~30%) the average area of the articular cartilage layer (Figure 6C) on the injured tibial joint surface compared to the untreated (PBS) group. Conversely, the *IL-1Ra* M2 MΦ treatment group significantly increased both the average width (Figure 6B) and average area (Figure 6C) of the articular cartilage layer by >25% and >40%, respectively, compared to the PBS (untreated) group. The articular cartilage histology-based OARSI OA score was accordingly reduced (Figure 6D). Perhaps due to the relatively large variations and small group size, the reduction in the OARSI OA score in the M2 MΦs alone group (i.e., GFP-expressing M2 MΦs group) was not significantly different from the untreated control group.

With respect to osteophyte formation, neither the *IL-1Ra*-expressing M2 MΦs nor the M2 MΦs group showed differences in the osteophyte maturity score (Figure 6E). Conversely, the meniscus score [a semi-quantitative measurement of the meniscal pathology associated with OA [49]] of the *IL-1Ra*-expressing M2 MΦ group, but not the M2 MΦs alone group, was reduced (Figure 6F). However, it did not reach the statistically significant level, perhaps due to the relatively large variations and the small group size. Regarding the synovial hypertrophy, the average thickness of the synovium layer of both the M2 MΦs treatment alone and the *IL-1Ra*-M2 MΦs treatment groups was significantly smaller than that of the untreated (PBS) injured tibial joints (Figure 6G), but the reduction in the *IL-1Ra* M2 MΦ treatment group appeared to be larger than that of the M2 MΦ alone group. Together, these findings indicate that the *IL-1Ra*-M2 MΦ-based adoptive gene transfer strategy is still effective in the reduction in or delay of OA progression when it was administered after OA would have been developed at 5 weeks post-injury.

## 4. Discussion

The concept of gene transfer-based strategies for OA/PTOA is not new, as many strategies utilizing direct in vivo injection of a recombinant viral vector to the articular cartilage or synovium [60,61] or ex vivo chondrocyte- or MSC-based gene transfer [62,63,64] approaches to deliver the therapeutic transgene to the synovium or articular cartilage have been attempted. These strategies, unfortunately, were mostly ineffective, in part because chondrocytes are present at a very low density or are located at varying depths with the dense matrix in the articular cartilage and are thereby inaccessible. Similarly, the chondrocyte- or MSC-based ex vivo gene therapies are unable to keep the genetically modified cells inside the injured joint long enough to yield sustained clinical benefits [62,63,64]. Other similar strategies utilizing bio-degradable scaffolds to deliver and maintain the therapeutic agents, viral vectors, or genetically modified cells to the OA joint [65,66] also lacked consistent efficacy as they are highly dependent on the bio-degradability and bio-compatibility of the scaffold(s). Accordingly, a pivotal requirement for successful adoptive cell or gene transfer therapy is the ability to target the vehicle cell to the specific disease tissue site combined with long-term retention in this tissue. Consequently, one of the principal potential reasons for the absence of long-term benefits for OA could be the limitation in the lack of an effective targeting mechanism for sustained and confined delivery of the therapeutic agent(s) to the afflicted joint.

We surmise that the use of M2 MΦs as the adoptive gene transfer cell vehicle platform could overcome this limitation and may allow for targeted, confined, and sustained delivery of the therapeutic transgene product to the inflamed OA joint. This concept is based on the fact that MΦs are rapidly recruited to and retained at sites of inflammation in response to systemic and local inflammation. We used M2 rather than M1 MΦs, because M2 MΦs are of anti-inflammatory and regenerative nature [31] and because an increase in M2 MΦs polarization improved OA symptoms [38], which is in contrast to the worsening of OA symptoms in response to an increase in M1 MΦ polarization [37,67]. *IL-1Ra* was chosen as the therapeutic gene because IL-1Ra is a promising disease-modifying OA drug [17,68,69,70]. Accordingly, the IL-1 receptor (IL-1R) is a key component of the NLR inflammasomes that mediates immunity and inflammatory responses [71] and triggers the degradation of articular cartilage in OA [16,20]. IL-1Ra is a specific inhibitor of IL-1 through the competitive binding for IL-1R against IL-1 [58] and thus blocks the IL-1R-mediated degradation of the articular cartilage, periarticular structure, and subchondral bone.

The present study presents three pieces of compelling evidence that the *IL-1Ra* M2 MΦ-based adoptive gene transfer approach for OA/PTOA is tenable. Firstly, CT bioluminescence imaging in vivo cell tracing and immunofluorescence histomorphometry confirm that the intraarticularly injected M2 MΦs were rapidly recruited to and retained exclusively at the inflamed synovia. That the transduced M2 MΦs were recruited to and retained at the inflamed joint is entirely consistent with our previous observation that systemically injected genetically modified M2 MΦs expressing vitamin D-1α-hydroxylase (*Cyp27b1*) were rapidly recruited to and retained at the inflamed bowel in a mouse model of inflammatory bowel disease [44]. The lenti-*IL-1Ra*-transduced M2 MΦs secreted substantial amounts of IL-1Ra protein. Therefore, it is conceivable that the recruited and retained genetically modified M2 MΦs could be a sustained local source of IL-1Ra to act on the afflicted joint to treat OA/PTOA.

This study also reveals that a large majority (~74%) of the injected M2 MΦs disappeared from the afflicted joint at 7 days after the local injection. The disappearance of these cells was likely due to their biological clearance, as the resolution of acute synovial inflammation during OA progression (usually occurs between 7 and 14 days after injury) triggers the clearance of synovial MΦs from inflamed sites that is mediated through either MΦs emigration to draining lymph nodes [72] or increased apoptosis of MΦs locally [73]. Because no detectable bioluminescence was seen in the lymph nodes, we tentatively conclude that the clearance of the injected M2 MΦs was mediated largely through local apoptosis. However, we cannot rule out the possibility that some of the cells were also cleared via lymph nodes through mass emigration. Despite the apparent rapid clearance of injected M2 MΦs, it should be emphasized that substantial numbers of the injected *Luc*-expressing M2 MΦs still remained exclusively at the inflamed synovium at 14 days after the cell injection, suggesting that the injected cells had a duration-of-stay inside the inflicted joint of at least 14 days. We did not extend the CT bioluminescence imaging in vivo cell tracing beyond 14 days. Therefore, our future studies will extend the tracing period for several weeks or even months to better define the duration-of-stay of the locally injected M2 MΦs, because such information is essential for the proper design and optimization of this M2 MΦ-based adoptive strategy for OA/PTOA.

Secondly, this study demonstrates that intraarticular injection of *IL-1Ra*-expressing M2 MΦs at 1 week post-injury appeared to be sufficient to yield an effective anti-OA response 3 weeks later. Accordingly, mice with an injection of *IL-1Ra*-expressing M2 MΦs at 1 week post-injury greatly reduced the plasma levels of COMP, increased the average area and width of articular cartilage at the tibial plateau, and decreased the OARSI OA score, when compared to mice receiving a single injection of GFP-expressing M2 MΦs (M2 MΦs alone control) or PBS (untreated control) at 4 weeks post-injury. Moreover, the *IL-1Ra*-M2 MΦs therapy reduced the average thickness of the synovium layer, indicating that the treatment appeared to also suppress the OA-associated synovial hyperplasia. This anti-synovial hyperplasia effect is consistent with the well-known anti-inflammatory action of IL-1Ra that suppresses synovitis in OA [58]. Together, these findings suggest strongly that early administration of the *IL-1Ra*-M2 MΦ-based adoptive gene transfer strategy is effective in preventing OA development or delaying OA progression.

Thirdly, this study shows that the *IL-1Ra*-expressing M2 MΦ-based gene transfer strategy, when it was administered at a time when OA has already been developed, appeared to also be efficacious. Accordingly, the *IL-1Ra*-M2 MΦs treatment administered at 5 weeks post-injury, at which time OA/PTOA is consistently developed in this mouse model [39,45,46], also decreased the plasma COMP level, reduced the OA-induced loss of articular cartilage, decreased synovial hyperplasia, and reduced the OARSI OA score, when compared to the GFP-expressing M2 MΦs control group and the untreated (PBS) group. However, it seems that the relative cartilage-sparing effects of the treatment when administered at later phases of OA development (i.e., 5 weeks post-injury) were relatively smaller than those of the treatment administered at the early phase (i.e., at 1 week post-injury). The reason for the smaller effects is unclear but it may be because there might be fewer *IL-1Ra*-expressing M2 MΦs to produce less amounts of IL-1Ra at the inflamed joint at later phases as opposed to those at the earlier phases of OA. In any event, the finding that this M2 MΦ-based adoptive gene transfer strategy is effective in animals with developed OA/PTOA is exciting and is clinically relevant, as patients with OA/PTOA usually do not seek medical advice until their disease is fully developed.

This study did not include an additional control group receiving only local injections of recombinant IL-1Ra protein for the comparison of the IL-1Ra protein therapy vs. adoptive gene transfer-based therapy, because our primary objective was to establish the feasibility of the M2 MΦ-based adoptive gene transfer platform and not to demonstrate the superiority of the M2 macrophage-based adoptive IL-1Ra gene transfer strategy over IL-1Ra protein therapy. It is also because the effect of treatment with high doses of recombinant IL-1Ra on OA has previously been investigated [74]. Nevertheless, that a single administration of the therapy is sufficient to modify OA/PTOA symptoms for at least 3 weeks is exciting and may also have clinical implications, since a single injection would be much more amenable to patients than the requirement of frequent multiple injections. It would also reduce any potential risks related to multiple injections, such as infections. In support of our speculation that a single administration of the M2 MΦs-based therapy could yield long-term clinical benefits, a previous study reported in a rat spinal cord injury model that a single application of the MΦ-based therapy initiated at 8 or 9 days post-injury produced sustained recovery effects even after 5 to 6 months [47]. However, because our investigation did not extend beyond four weeks, it remains to be confirmed as to whether a single cell injection could indeed yield long-term and sustainable clinically relevant benefits.

Synovial MΦs, particularly during synovitis, are known to release TGF-β and related growth factors to promote the formation and maturation of osteophytes [8], and transgenic overexpression of IL-1Ra in rabbits with OA has been shown to reduce the osteophyte size [69]. Accordingly, our finding that this *IL-1Ra*-M2 MΦ-based adoptive gene transfer strategy, while effectively protecting articular cartilage degradation, did not seem to have protective effects on osteophyte formation/maturation as determined by the osteophyte maturity score is unexpected and puzzling. We currently do not have an explanation, and we also cannot rule out the possibility that this *IL-1Ra*-M2 Φ-based adoptive gene transfer treatment might not be able to ameliorate the degenerative changes in osteophyte and meniscus in our tibial plateau injury-induced model of OA/PTOA. Understanding of the mechanistic cause for the lack of a suppressive effect of the *IL-1Ra*-M2 MΦs therapy on osteophyte formation could offer important insights into the regulatory role of IL-1β and IL-1Ra on osteophyte formation. Consequently, we will in the future investigate whether this therapy would be able to reduce/prevent the OA-induced osteophyte formation and meniscal deterioration with a different animal model of OA/PTOA, such as surgical destabilization of the medial meniscus (DMM).

The IL-1Ra-based therapy is considered an anti-catabolic therapy for OA/PTOA, since its primary function in OA is to inhibit the IL-1-mediated degradation of articular cartilage. Accordingly, the relatively large increases in the area and width of the articular cartilage layer in response to both the *IL-1Ra*-M2 MΦs and to the *GFP*-M2 MΦs alone treatment were somewhat unexpected. They may raise the intriguing possibility that the M2 MΦ-based treatment also has an anabolic action on articular cartilage regeneration. In this regard, M2 MΦs per se have shown disease-modifying functions in inflammatory diseases, including OA, due to their anti-inflammatory properties that potentially alleviate articular cartilage damage and intraarticular inflammation as well as to their ability to suppress the destructive effects of pro-inflammatory M1 MΦs within the inflamed joint space [38,57]. It has also been documented that M2 MΦs participate in tissue repair through their secretion of numerous cartilage growth factors, such as IGF-I, FGF-b, PDGF-b, and cartilage-derived BMPs after the acute inflammatory phase [30,36]. It is conceivable that these M2 MΦs-derived anabolic growth factors might participate in the M2 MΦ-dependent regeneration of articular cartilage processes, which might be responsible for the observed anabolic effects. If confirmed, the use of M2 MΦs as the cell vehicle could offer an additional advantage (i.e., an anabolic action in addition to its documented anti-catabolic action) for this M2 MΦ-based adoptive gene transfer platform over the other types of gene transfer-based platforms as a therapeutic strategy for OA/PTOA.

Lastly, this strategy could easily be adapted to an autologous gene transfer strategy by using the patient’s own blood-derived M2 MΦs, since there are established methods for the isolation of large numbers of M2 MΦs from circulating blood [75]. The use of a patient’s own blood-derived MΦs has a key advantage as it can avoid immune issues associated with non-compatibility. The M2 MΦs-based strategy might also have an additional theoretical advantage, in that it affords a regulated targeting mechanism for OA therapy with a unique in vivo on–off switch: the therapeutic M2 MΦs are recruited to OA synovia when they are inflamed and are dissipated when the inflammation subsides.

This M2 MΦ-based adoptive gene transfer strategy, however, has several limitations: (1) the number of mice in each test group was relatively small (i.e., *n* = 6–7 per group). The group size was chosen based on our past studies of similar nature, because there was insufficient information for a proper power analysis at the time. Accordingly, it is possible that this group size might not be sufficient to detect the smaller differences, especially in the presence of high variations. Therefore, we will in our future studies confirm key findings with an increased group size that has sufficient power to detect significant differences in the relevant parameters. (2) This feasibility study was performed exclusively with male mice. However, we acknowledge that sex is an important risk factor for OA/PTOA [3,4]. Thus, our future studies must confirm the feasibility of this IL-1Ra/M2 MΦ-based adoptive gene transfer strategy in female mice. (3) The use of the lentiviral vectors to transduce M2 MΦs could have intrinsic problems that include the following: (i) the number of transgene copies could vary significantly in resulting transduced M2 MΦs; (ii) lentiviral transduction also causes variable cell death; and (iii) the insertion of a transgene in the genome of transduced macrophages is relatively random, which could potentially alter, but differently, their proliferation and survival. These issues could introduce significant variables, confounding factors, and safety issues. However, we believe that this limitation could be overcome with the use of the CRISPR/Cas9 technology to insert the therapeutic transgene into M2 MΦs. The CRISPR/Cas9 technology has been matured to the stage that it could readily be adopted for this and other similar adoptive gene transfer strategies. (4) Both the control and *IL-1Ra*-expressing M2 MΦs were manipulated with the lentiviral transduction and expressed GFP protein. The viral transduction process and/or GFP expression per se could alter cellular functions that could lead to ambiguous and/or misleading results. To rule out this possibility, we may in the future include an un-transduced wild-type M2 MΦ group as an additional control for comparison. (5) M2 MΦs used in this study were isolated through the in vitro differentiation, polarization, and expansion approach and have not been purified, e.g., with flow cytometry sorting or magnetic-activated cell sorting (MACS). Accordingly, despite our flow cytometry analysis, based solely on the expression of an M2 MΦ surface marker, i.e., CD206, indicates that the purity of our M2 MΦ preparations was 85–90%, we cannot rule out the unlikely possibility that the observed effects were caused by contaminated cells of monocytic lineage. Nevertheless, because MΦs, including M2 MΦs, exhibited a high degree of heterogeneity and plasticity [30,32], these findings must be confirmed with more purified and better-characterized M2 MΦs preparations (e.g., with other M2 surface markers) to ensure that the therapeutic effects were indeed attributable to a specific M2 MΦ phenotype.

## 5. Conclusions

This study provides strong evidence for the feasibility of the use of M2 MΦ as the cell vehicle platform for the M2 MΦ-based adoptive gene transfer therapy for OA/PTOA. If this proof-of-principle feasibility study is confirmed and extended by further evidence that this strategy can effectively (1) reduce inflammation at the injured joint, (2) prevent degradation of articular cartilage and subchondral bone erosion, and (3) promote regeneration of articular cartilage, it would offer compelling rationale for the further development and optimization of such an innovative strategy. If successful, this therapy could translate into enormous benefits both in terms of savings in medical dollars for managing this disease and in human suffering.

## Data Availability

The research data that support the findings of this study are stored at an approved storage facility within the Loma Linda VA Healthcare system and are available for review upon reasonable request and approval by the Loma Linda VA Healthcare system.
